# Species diversity in the new lamprey genus *Occidentis*, formerly classified as western North American ‘*Lampetra*’

**DOI:** 10.1371/journal.pone.0313911

**Published:** 2024-12-19

**Authors:** Kellie J. Carim, Grace Auringer, Margaret F. Docker, Claude B. Renaud, Benjamin J. Clemens, Monica R. Blanchard, Christina Parker, Michael K. Young

**Affiliations:** 1 Aldo Leopold Wilderness Research Institute, Rocky Mountain Research Station, U.S. Forest Service, Missoula, Montana, United States of America; 2 Department of Animal Science, University of California, Davis, California, United States of America; 3 Department of Biological Sciences, University of Manitoba, Winnipeg, Manitoba, Canada; 4 Research and Collections, Canadian Museum of Nature, Ottawa, Ontario, Canada; 5 Corvallis Research Lab, Oregon Department of Fish and Wildlife, Corvallis, Oregon, United States of America; 6 Washington Department of Fish and Wildlife, Ridgefield, Washington, United States of America; 7 California Department of Fish and Wildlife, Sacramento, California, United States of America; 8 National Genomics Center for Wildlife and Fish Conservation, Rocky Mountain Research Station, U.S. Forest Service, Missoula, Montana, United States of America; Oklahoma State University, UNITED STATES OF AMERICA

## Abstract

Accurate taxonomy is fundamental to the study and conservation of biodiversity. Because of their morphological similarities, most brook and river lampreys in western North America have been placed in the genus *Lampetra* along with lampreys from Eurasia and eastern North America. However, molecular-based phylogenetic studies dating back several decades indicate that lampreys from Pacific drainages are genetically distinct from Atlantic *Lampetra*. Reviewing previous phylogenetic analysis of two mitochondrial and two nuclear genes for Northern Hemisphere lampreys, we assign these western North American brook and river lampreys to a new genus, *Occidentis*. To assess species diversity within *Occidentis*, we performed a species delimitation analysis using all publicly available cytochrome *b* sequences of the genus. Similar to previous studies, *O*. *ayresii* and *O*. *richardsoni* were not reciprocally monophyletic and are best categorized as life history variants of a single species. In addition to *O*. *pacifica*, *O*. *hubbsi*, and the diverse *O*. *ayresii* species complex, as many as seven undescribed candidate species from Oregon and California were identified, supporting results from previous studies with more geographically limited datasets. One specimen from Paynes Creek, California, was identified as a candidate species, although this single individual showed minimal interspecific divergence (1.34%) with *O*. *hubbsi*. Further genetic assessment along with information on morphology and phylogeography is needed to determine whether the variation observed between groups of candidate species represents distinct species or divergent lineages within a species complex. Additional sampling will inform whether there are additional species not currently represented in this dataset. Thus, the number of species formally recognized under *Occidentis* is subject to change with new information. Systematic assessment of the distribution and phylogenetic complexity within *Occidentis* will enhance our understanding of its evolutionary history and taxonomic diversity, which will guide efforts to conserve the biodiversity of lampreys.

## Introduction

Accurate taxonomy is fundamental to systematics and biogeography, and thus to conservation of biodiversity [1, 2]. In recent years, phylogenetic examinations based on molecular characters for several groups of fishes have revealed taxonomic relationships that differ from widely accepted classifications based solely on morphology [3]. These taxonomic inconsistencies are apparent among lampreys in the order Petromyzontiformes [4–6]. For example, molecular analyses have demonstrated that some genera are para- or polyphyletic [5, 7, 8] and reveal new information regarding the diversity and placement of species within genera [9, 10]. In addition, a common pattern among genera is the presence of species complexes that are described as paired or stem-satellite species [11]. Each complex consists of one species that exhibits predatory and migratory behavior as an adult and one or more related species that are non-migratory and non-feeding as adults [11, 12]. However, the members of these complexes are indistinguishable at the larval stage [13]. At present, most of these forms are regarded as separate taxa, but molecular data have raised doubts about this interpretation [14]. These issues are evident among members of the genus *Lampetra*, in which taxonomic designations have long been unstable and contentious (reviewed by [9, 15]).

*Lampetra* was originally proposed by Bonnaterre, 1788 [16] but without any associated species. Fowler (1925) [17] established European river lamprey *Petromyzon fluviatilis* Linnaeus, 1758 [18] as the type species of this genus, resulting in reclassification of the species to *Lampetra*. Subsequent classifications of species within this genus have fluctuated. At its peak, some authorities [19] included *Lethenteron* Creaser & Hubbs, 1922 [20], *Entosphenus* Gill, 1862 [21], and even *Tetrapleurodon* Creaser & Hubbs, 1922 [20] and *Eudontomyzon* Regan, 1911 [22, 23] as subgenera of *Lampetra*, so that the majority of Northern Hemisphere lamprey species were included in this genus. However, a cladistic study using morphological characters supported separate generic designation of *Lethenteron*, *Entosphenus*, *Tetrapleurodon*, and *Eudontomyzon* [24].

Taxonomic treatments of lampreys based on morphological traits generally recognize 12 species of *Lampetra*. Of these, eight species are distributed in Eurasia: *L*. *fluviatilis*; European brook lamprey *L*. *planeri* (Bloch, 1784) [25]; Turkish brook lamprey *L*. *lanceolata* Kux & Steiner, 1972 [26]; Po brook lamprey *L*. *zanandreai* Vladykov, 1955 [27]; Costa de Prata lamprey *L*. *alavariensis* Mateus et al., 2013 [28]; Nabão lamprey *L*. *auremensis* Mateus et al., 2013 [28]; Sado lamprey *L*. *lusitanica* Mateus et al., 2013 [28], and Šoljan’s brook lamprey *L*. *soljani* Tutman et al., 2017 [29]. The remaining four species are distributed in North America, three of which are in western North America: western river lamprey *L*. *ayresii* (Günther, 1870) [30]; western brook lamprey *L*. *richardsoni* Vladykov & Follett, 1965 [31]; and Pacific brook lamprey *L*. *pacifica* Vladykov, 1973 [32]. Least brook lamprey *L*. *aepyptera* (Abbott, 1860) [33] is the sole member of the genus distributed in eastern North America (see also [12]).

The relationships among *Lampetra* species have been assessed primarily through similarities in adult morphology and feeding habits following metamorphosis [12, 24]. For example, the migratory, parasitic western river lamprey *L*. *ayresii* was synonymized with the migratory, parasitic European river lamprey *L*. *fluviatilis* by Regan (1911) [23]. However, Vladykov & Follett (1958) [34] redescribed *L*. *ayresii* as a distinct species based on slight differences in body proportions, caudal fin shape, pigmentation, and average trunk myomere count. A cladistic analysis of morphological characters limited to parasitic lampreys [24] determined that *L*. *fluviatilis* and *L*. *ayresii* constituted a clade sharing two synapomorphies: the presence of tricuspid lateral circumoral teeth and the absence of velar wings. Similarly, the western brook lamprey *L*. *richardsoni* and European brook lamprey *L*. *planeri* are both freshwater-resident and nonparasitic and show only subtle morphological differences from each other [31]. Based on common geographic distributions and larval traits, but differing adult phenotypes and life histories, *L*. *richardsoni* and *L*. *ayresii* were considered paired species, as were *L*. *planeri* and *L*. *fluviatilis* [11, 12].

**Fig 1:**
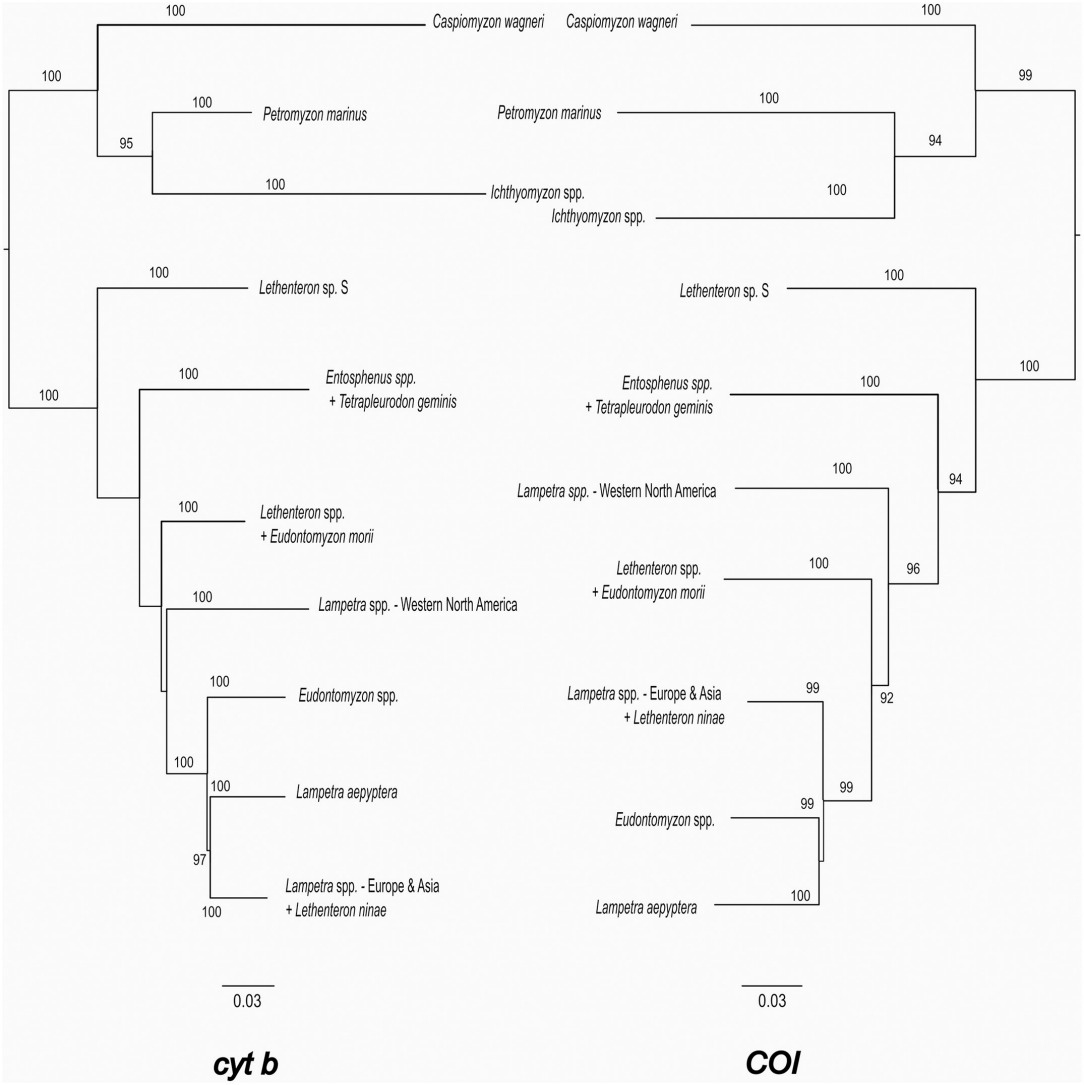
Maximum likelihood tree showing the phylogenetic relationships of Northern Hemisphere lampreys at the *cyt b* and COI genes, condensed to the genus level, reproduced from Carim et al. [36]. Bootstrap values are shown for branches with > 85% support. The branching patterns show a polyphyletic relationship among members formally designated as *Lampetra*.

While morphological features are highly useful in delineating taxa, DNA sequence variation has emerged as another powerful tool in taxonomic studies, especially for lampreys that lack the bony structures typically used to distinguish fish taxa. In contrast to morphology-based classification [24], phylogenetic studies based on molecular characters have repeatedly demonstrated para- and polyphyletic relationships within and among species of *Lampetra* (Table 1) [4, 5, 7–9, 35, 36] and the existence of unrecognized, cryptic species [36–39]. For example, molecular evidence demonstrates that *L*. *ayresii* and *L*. *richardsoni* are not reciprocally monophyletic [38] and appear to be best characterized as life history variants of a single species [36]. Species delimitation analyses have also revealed the presence of several potentially undescribed cryptic species in western North America [36–38] and reduced the originally proposed distribution of *L*. *pacifica* [36, 40]. Additionally, several phylogenetic studies further explore the membership of this genus. For example, molecular data [7, 36, 41] suggested that the western Transcaucasian brook lamprey *Lethenteron ninae* Naseka et al., 2009 [42] likely represents a ninth *Lampetra* species from Eurasia (see also [43]; Table 1, Fig 1). Similarly, the Kern brook lamprey *L*. *hubbsi* (Vladykov & Kott, 1976) [44] of western North America was originally classified as *Entosphenus*, but molecular information resulted in reclassification to *Lampetra* [5, 9]. Conversely, other molecular studies have questioned whether *L*. *aepyptera* is truly a member of *Lampetra* [5, 36; see also 7]. In a comprehensive molecular analysis of the cytochrome *b* (*cyt b*) gene among members of the Petromyzontidae family, Carim et al. [36] showed *L*. *aepyptera* as sister to a clade comprising Eurasian *Lampetra* plus *Le*. *ninae*, and western North American *Lampetra* as sister to a clade that includes the former two clades plus *Eudontomyzon* (Fig 1). In the same study, comprehensive analysis at a second mitochondrial gene, cytochrome oxidase subunit I (COI), showed polyphyly among the same groups, with slight variation in placement of sister taxa (Fig 1). Li [7] performed a phylogenetic analysis to examine the relationships among *Lethenteron* and its most closely related taxa (*Eudontomyzon* and *Lampetra*) at two nuclear genes (transponder associated with antigen processing and SRY-related high mobility group box D, or TAP2 and soxD, respectively). Results of this study further supported the relationships revealed by *cyt b* and COI analyses, including separation of *Lampetra* from western North America, eastern North America, and Eurasia (including *Le*. *ninae*) as distinct taxonomic groups [7]. To resolve the lack of monophyly, Lang et al. [5] suggested assigning *L*. *aepyptera* to its own genus, *Okkelbergia*, which was originally designated as a subgenus of *Lampetra* by Creaser & Hubbs (1922) [20] (see also [15, 19]). However, retaining western North American river and brook lampreys as members of *Lampetra* remains problematic and obscures the phylogenetic distinctiveness of this group of lampreys relative to Eurasian *Lampetra*. Further work is also needed at the species level to identify cryptic species (e.g., [38, 45]) and to examine, on a case-by-case basis, whether the so-called species pairs (or in some cases, satellite species *sensu* [46]) represent distinct taxa or phenotypic variants of a single taxon [47].

**Table 1:** Summary of the taxonomy of *Lampetra* and related groups discussed in this study, as determined by morphologically based studies. Under "Authority", citations in parentheses indicate species that have been assigned to a genus different from their original description. Note that this study erects a new genus for *Lampetra* of western North America. Updated from Docker et al. [9] and Potter et al. [15].

	Scientific name	Authority	Common name	Life history	Distribution	Notes
**Europe and Asia**						
	*Lampetra fluviatilis*	(Linnaeus, 1758)	European river lamprey	Migratory, parasitic	Drainages of northeastern Atlantic Ocean	Considered a species pair with *L*. *planeri*. Originally classified by Linnaeus (1758) [18] as *Petromyzon fluviatilis*.
	*Lampetra planeri*	(Bloch, 1784)	European brook lamprey	Resident, nonparasitic	Similar to *L*. *fluviatilis*, plus Danube and Volga river drainages	Considered a species pair with *L*. *fluviatilis*. Originally classified by Bloch (1784) [25] as *Petromyzon planeri*.
	*Lampetra lanceolata*	Kux & Steiner, 1972	Turkish brook lamprey	Resident, nonparasitic	Iyidere River, Turkey	
	*Lampetra zanandreai*	Vladykov, 1955	Po brook lamprey	Resident, nonparasitic	Drainages of the Adriatic Sea	Sometimes assigned to *Lethenteron* because posterior circumorals are present in most specimens (e.g. [48, 49]), but the original generic assignment is consistent with molecular data [15].
	*Lampetra auremensis*	Mateus et al., 2013	Nabão lamprey	Resident, nonparasitic	Tagus River drainage, Portugal	
	*Lampetra lusitanica*	Mateus et al., 2013	Sado lamprey	Resident, nonparasitic	Sado River drainage, Portugal	
	*Lampetra alavariensis*	Mateus et al., 2013	Costa de Prata lamprey	Resident, nonparasitic	Esmoriz and Vouga River drainages, Portugal	
	*Lampetra soljani*	Tutman et al., 2017	Šoljan’s brook lamprey	Resident, nonparasitic	Neretva River, Adriatic Sea basin	
	*Lethenteron ninae*	Naseka et al., 2009	Western Transcaucasian brook lamprey	Resident, nonparasitic	Drainages of the Black Sea	Still formally classified under *Lethenteron* [15, 50]. Reclassification to *Lampetra* supported by molecular data [7, 36, 41].
**Eastern North America**						
	*Lampetra aepyptera*	(Abbott, 1860)	Least brook lamprey	Resident, nonparasitic	Drainages of northwestern Atlantic Ocean and Gulf of Mexico	Based on dentition, Hubbs & Potter [19] assigned this species to a new genus, *Okkelbergia*, which was originally erected as a subgenus of *Lampetra* for this species by Creaser & Hubbs [20]; although it forms a monophyletic group with Eurasian *Lampetra*, genus-level distinctiveness is supported by molecular data [5, 36].
**Western North America**						
	*Lampetra ayresii*	(Günther, 1870)	Western river lamprey	Migratory, parasitic	Drainages of eastern Pacific from Alaska to California	Considered a species pair with *L*. *richardsoni*. Synonymized with *L*. *fluviatilis* by Regan [23] but redescribed as *L*. *ayresii* by Vladykov & Follett [34] based on slight differences in body proportions, caudal fin shape, pigmentation, and average trunk myomere count.
	*Lampetra richardsoni*	Vladykov & Follett, 1965	Western brook lamprey	Resident, nonparasitic	Drainages of eastern Pacific from Alaska to California	Considered a species pair with *L*. *ayresii*. Described as distinct from *L*. *planeri* by Vladykov & Follett (1965) [31] based on subtle differences in dentition, body proportions, and pigmentation. Carim et al. [36] synonymized *L*. *richardsoni* with *L*. *ayresii* based on a lack of genetic differentiation (see also [38]).
	*Lampetra pacifica*	Vladykov, 1973	Pacific brook lamprey	Resident, nonparasitic	Columbia River drainages, Oregon	Formerly synonymized with *L*. *richardsoni* by Robins et al. [51] but validated as distinct by Reid et al. [40]. Vladykov (1973) [32] estimated a broad distribution including in Sacramento and San Joaquin river systems in California, but Reid et al. [40] restricted *L*. *pacifica* to the Columbia River system.
	*Lampetra hubbsi*	(Vladykov & Kott, 1976)	Kern brook lamprey	Resident, nonparasitic	Friant-Kern Canal and Merced River, California	Originally placed in the genus *Entosphenus* based on dentition, but molecular data (e.g., [5, 9, 38]) unambiguously placed the species in *Lampetra* with other western North American lampreys and it is now widely recognized as such (e.g., [15, 52]).

To formally recognize the distinctiveness of these western North American lamprey and resolve the polyphyletic relationships observed in the current taxonomic classification, we reclassify this group to a new genus. There is no available genus name to which these species from western North America can be assigned [53]; therefore, we propose the name *Occidentis*, gen. nov. *urn*:*lsid*:*zoobank*.*org*:*act*: *FF175211-167B-4FFD-8499-9F6AC2400164*. We fix the type species of this new genus to be *Petromyzon ayresii* Günther, 1870 [30].

### Etymology

*Occidentis* is the feminine genitive declension of the adjective *occidens* and translates to “westerly” or “of the west”. This name refers to the broad geographic distribution of the genus across western North America, which is geographically separate from *Lampetra* of eastern North America and *Lampetra* of Eurasia (including *Lethenteron ninae*).

### Diagnosis

According to the phylogenetic tree derived from *cyt b* sequences in Carim et al. [36], the new genus is distinguishable from its nearest generic relatives *Eudontomyzon* and *Lampetra* of eastern North America and *Lampetra* of Eurasia (including *Lethenteron ninae*; Fig 1) by fixed nucleotide differences at the following positions (relative to nucleotide positions in the complete mitochondrial sequence of GenBank accession Y18683 of *Lampetra fluviatilis*): 12494, 12557, 12683, 12758, 12764, 12821, 12890, 12902, 12935, 12962, 12998, 13007, and 13136 (S1 Table).

The adult or metamorphosed stage of *Occidentis* has two dorsal fins, and the oral disc possesses two unicuspid teeth (rarely three in *O*. *hubbsi*; see [44, 54]) on the supraoral lamina, but no exolateral teeth. *Eudontomyzon* and *Lampetra* from eastern North America are distinguishable from *Occidentis* by the presence of 1–6 rows of exolateral teeth [43]. *Lampetra* from Eurasia including *Lethenteron ninae* are not distinguishable morphologically from *Occidentis* [28, 29, 43] but each is restricted to a portion of a separate continent (western Eurasia vs. western North America) and ocean basin (North Atlantic vs. North Pacific) in (see Table 1).

### Nomenclatural acts

The electronic edition of this article conforms to the requirements of the amended International Code of Zoological Nomenclature, and hence the new names contained herein are available under that Code from the electronic edition of this article. This published work and the nomenclatural acts it contains have been registered in ZooBank, the online registration system for the ICZN. The ZooBank LSIDs (Life Science Identifiers) can be resolved and the associated information viewed through any standard web browser by appending the LSID to the prefix “http://zoobank.org/”. The LSID for this publication is: urn:lsid:zoobank.org:pub: FF175211-167B-4FFD-8499-9F6AC2400164. The electronic edition of this work was published in a journal with an ISSN, has been archived and is available from the following digital repositories: LOCKSS and the United States Forest Service Treesearch (https://www.fs.usda.gov/research/treesearch).

In this paper, we complement the maximum likelihood trees generated by Carim et al. [36] with an additional analysis (genetic distance analysis among Petromyzontidae) to further support the genetic distinctiveness of *Occidentis* from *Lampetra* of eastern North America and Eurasia. To more comprehensively describe the diversity within this newly named genus, we combine data from two recent studies [36, 37], which include sequence data from several previous works [5, 38, 40, 55], to perform a formal species delimitation analysis. This analysis expands upon previous work (e.g., [36]) by putting novel lineages observed by Auringer et al. [37] into the context of all *Occidentis*, expanding our knowledge of the diversity within and among candidate species of this newly named genus.

## Methods

### Genetic distances among Petromyzontidae

To complement previous phylogenetic work [4, 5, 7–9, 35, 36] distinguishing *Occidentis* as separate from *Lampetra* of eastern North America and Eurasia, we calculated genus-level pairwise distance matrices for members of Petromyzontidae at the *cyt b* and COI genes. The *cyt b* dataset (trimmed to 822 bp) includes all sequences of Petromyzontidae used by Carim et al. [36] to generate the *cyt b* maximum likelihood tree in Fig 1 (*n* = 428), as well as an additional 109 sequences of *Entosphenus* and *Occidentis* made publicly available after that publication (S2 and S3 Tables; S1 File; [37, 55]). The COI dataset included 481 sequences trimmed to 585 bp (S3 Table). Note that the COI dataset was identical to sequences of Petromyzontidae used by Carim et al. [36] to generate the COI maximum likelihood tree in Fig 1, as there are no new publicly available COI sequences since that publication. We used MEGA 7 [56] to calculate pairwise genetic distances among all sequences, expressed as p-distance, and identified the minimum intergeneric and maximum intrageneric pairwise genetic distances. Sequences were grouped by genus level based on in Fig 1, including separation of *Lethenteron* sp. S into its own genus (see [7, 10]). While Fig 1 collapses the clade containing *Tetrapleurodon* and *Entosphenus* for ease of viewing, we treat these as distinct genera because they are reciprocally monophyletic in both genetic and cladistic analyses [24, 36], and no work has proposed combining the two.

### Species delimitation analysis

For the species delimitation analysis, we used all *cyt b* sequences in GenBank for *Occidentis* (*n* = 192 sequences representing 305 individuals; S2 Table; see also S1 File). Codon positions aligning to amino acids were identified, and sequences were condensed to 66 representative haplotypes (S2 Table).

Following Carim et al. [36], we used five methods of single-locus species delimitation. We recognized species under the phylogenetic species concept, which relies on reciprocal monophyly among lineages [57]. For stringency, we modified this concept by requiring a minimum distance threshold of 2% among candidate species, which is often typical of interspecific differences among fishes [58]. We further assessed the results for consistency among the five methods. We acknowledge that other species concepts or practitioners with a different background might suggest a different interpretation of species boundaries (*sensu* [59]). However, we regard our designations as conservative and consistent in aligning the present taxonomy with evolutionary patterns.

To prepare data for the species delimitation analyses, we estimated a maximum likelihood tree in IQ-TREE 2.1.1 [60] implemented via the Cyberinfrastructure for Phylogenetic Research (CIPRES) gateway (https://www.phylo.org/). Prior to constructing the tree, we assigned three preliminary partitions based on codon position (see [61]), then selected edge-linked partitions and the TESTMERGE setting to determine the best-fitting evolutionary model (as measured by BIC) for each partition. The estimated best fit evolutionary model for analysis was K2P+1 for codon positions one and two, and TN+F+G3 for codon position three. We used Pacific lamprey *Entosphenus tridentatus* (Gairdner in Richardson, 1836) [62] as an outgroup (GenBank accession GQ206157) and estimated branching patterns with 1000 ultrafast bootstraps. The final trees were visualized and edited using FigTree v1.4.4 (http://tree.bio.ed.ac.uk/software/figtree) and Inkscape v1.0.1 (http://inkscape.org).

For the first species delimitation analysis, the maximum likelihood tree was input into the online version of Poisson tree processes (PTP; https://mptp.h-its.org/#/tree; [63]), which examines branch lengths to estimate the shift from intraspecific to interspecific divergence and can provide a liberal estimate of species diversity relative to other methods [64]. For the PTP analysis, we used the single-rate option with the default significance setting. Second, we constructed a statistical parsimony network (SPN) with a 97% parsimony limit in TCS v.1.21 [65] and visualized these using tcsBU (https://cibio.up.pt/software/tcsBUl; [66]). When applied to datasets with multiple taxa, the number of unconnected networks constitutes a conservative estimate of species diversity [67]. Third, we used the online version of Assemble Species by Automatic Partitioning (ASAP; https://bioinfo.mnhn.fr/abi/public/asap/#; [68]), for which pairwise genetic distances among haplotypes are used to distinguish between intraspecific variation and interspecific divergence, and a scoring system identifies the best-fitting set of partitions (i.e., candidate species) based on both the probability of panmixia in a given partition and genetic distances within and among partitions. The set of partitions with the lowest score is deemed the most likely number of species. We adopted the default values and distance set at 2.0 based on the K80 substitution model because of its similarity to the traditional distance metric used in sequence-based analyses [69]. The analysis was run 10 times with different initial seeds, for which the highest-scoring set of partitions only changed once. Because the approach is similar to ABGD [70], we assumed that species counts would be conservative relative to other methods [68]. Following the PTP, SPN, and ASAP analyses, we reexamined the maximum likelihood tree from the first step and interpreted bootstrap values ≥ 85 as acceptable support [71] for a candidate species identified by one of the preceding methods. For the fifth and final method of species delimitation, we used MEGA 7 [56] to examine pairwise genetic distances, expressed as p-distance, among the candidate species identified by the above methods. We contrasted maximum intraspecific distances with minimum interspecific distances. If the latter was near or exceeded 2% and a barcode gap was present [69], we interpreted this as supporting the designation of a candidate species. Following Carim et al. [36], we treated all specimens originally labeled *L*. *richardsoni* as representatives of *L*. *ayresii*.

## Results

### Genetic distances among Petromyzontidae

We have proposed the name *Occidentis*, gen. nov. *urn*:*lsid*:*zoobank*.*org*:*act*: *FF175211-167B-4FFD-8499-9F6AC2400164* to recognize the genetic distinctiveness of western North American brook and river lampreys formally classified at *Lampetra*. The minimum pairwise genetic distances among genus-level groupings ranged from 3.40–16.20% and 3.08–14.87% at the *cyt b* and COI genes, respectively (Tables 2 and 3; see also S4 and S5 Tables). Minimum intergeneric pairwise distances between *Occidentis* and *Lampetra* of eastern North America and Eurasia exceeded 6% at both genes, with a substantial barcode gap at the COI gene (Tables 2 and 3).

**Table 2:** Genetic differences (measured as p-distance and expressed as a percentage) within and among genera of the Petromyzontidae family at the cyt *b* gene. Values on the diagonal (in bold) are maximum intrageneric differences; values below the diagonal are minimum intergeneric differences. Members of *Tetrapleurodon* were represented by a single haplotype, and therefore the intrageneric difference could not be estimated.

	*Caspiomyzon*	*Petromyzon*	*Ichthyomyzon*	*Lethenteron* sp. S	*Entosphenus*	*Tetrapleurodon*	*Lethenteron + Eudontomyzon morii*	*Occidentis*	*Eudontomyzon*	*Lampetra aepyptera*	*Lampetra + Lethenteron ninae*
*Caspiomyzon*	**10.10**										
*Petromyzon*	11.80	**0.24**									
*Ichthyomyzon*	15.50	12.40	**9.60**								
*Lethenteron* sp. S	14.00	13.38	15.00	**2.31**							
*Entosphenus*	12.70	12.30	15.30	11.20	**0.90**						
*Tetrapleurodon*	13.10	12.90	15.10	11.10	3.40	**—**					
*Lethenteron + Eudontomyzon morii*	13.60	12.29	14.50	9.98	8.60	9.50	**3.89**				
*Occidentis*	12.80	12.29	14.50	9.12	7.40	8.60	6.33	**6.81**			
*Eudontomyzon*	13.70	12.40	15.90	9.10	8.20	9.10	6.10	5.80	**3.60**		
*Lampetra aepyptera*	14.20	12.77	16.20	10.22	9.00	10.20	6.08	6.93	4.10	**4.87**	
*Lampetra + Lethenteron ninae*	13.90	12.90	16.20	9.10	8.50	9.40	6.10	6.30	3.40	3.80	**4.40**

**Table 3:** Genetic differences (measured as p-distance and expressed as a percentage) within and among genera of the Petromyzontidae family at the COI gene. Values on the diagonal (in bold) are maximum intrageneric differences; values below the diagonal are minimum intergeneric differences.

	*Caspiomyzon*	*Petromyzon*	*Ichthyomyzon*	*Lethenteron* sp. S	*Entosphenus*	*Tetrapleurodon*	*Lethenteron + Eudontomyzon morii*	*Occidentis*	*Eudontomyzon*	*Lampetra aepyptera*	*Lampetra + Lethenteron ninae*
*Caspiomyzon*	**8.55**										
*Petromyzon*	12.48	**0.51**									
*Ichthyomyzon*	9.74	11.28	**7.01**								
*Lethenteron* sp. S	11.28	12.99	12.65	**2.22**							
*Entosphenus*	11.28	13.50	11.62	8.72	**0.86**						
*Tetrapleurodon*	13.16	14.36	12.99	10.77	3.08	**0.17**					
*Lethenteron + Eudontomyzon morii*	13.50	14.87	13.68	10.26	9.06	9.92	**2.22**				
*Occidentis*	11.62	14.53	13.33	9.57	7.69	9.40	7.69	**3.76**			
*Eudontomyzon*	11.11	14.53	11.28	9.23	7.52	9.40	7.52	6.84	**4.44**		
*Lampetra aepyptera*	11.62	13.85	11.80	9.74	8.03	9.40	7.35	6.50	3.42	**4.27**	
*Lampetra + Lethenteron ninae*	11.45	13.68	11.45	8.55	7.86	9.23	6.67	7.01	3.42	3.93	**3.25**

### Species delimitation within *Occidentis*

**Fig 2:**
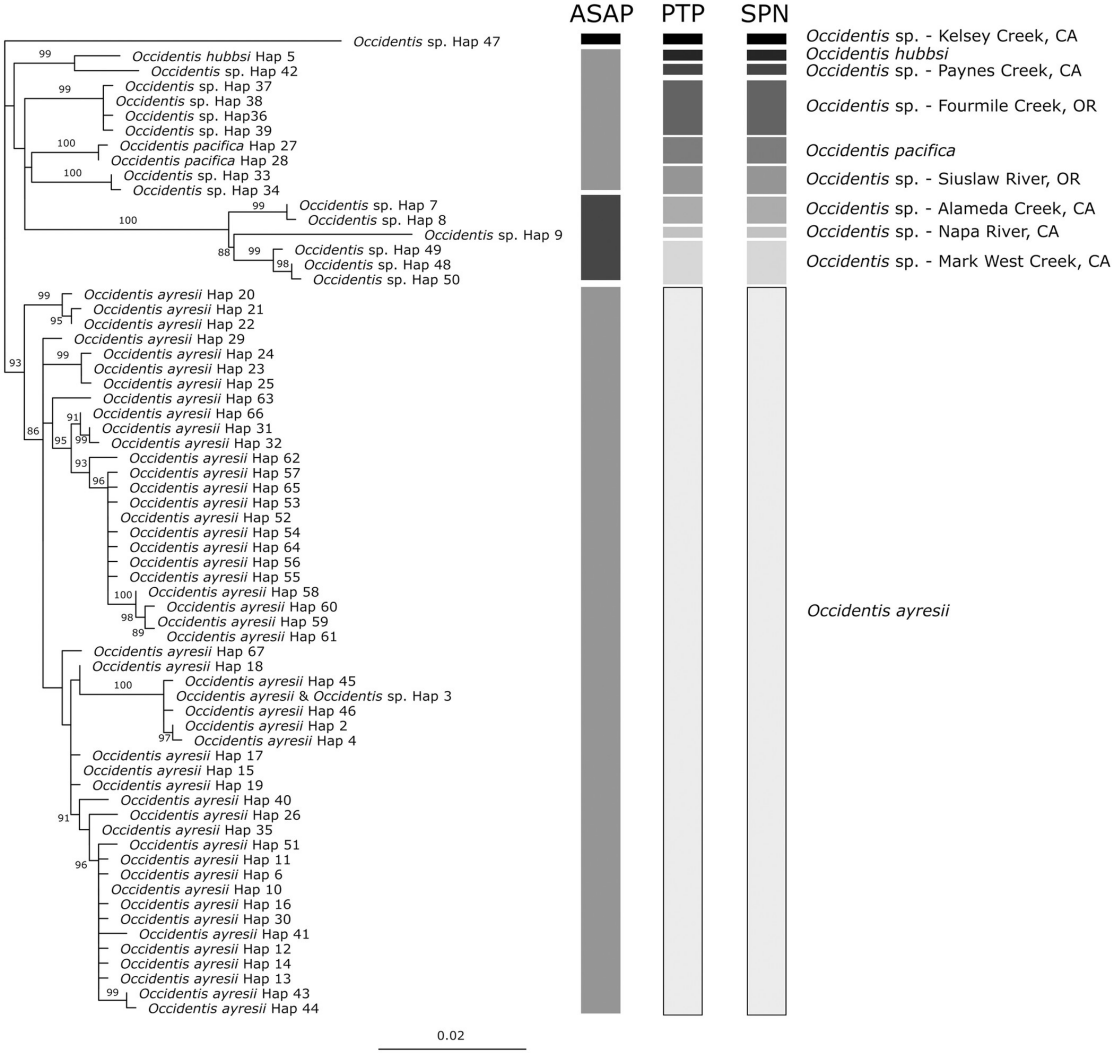
Maximum likelihood tree displaying relationships among *Occidentis* specimens at the *cyt b* gene. Tip labels correspond to *cyt b* haplotypes in S2 Table. Bootstrap values are shown for branches with > 85% support. Bars to the right of the phylogenetic tree denote species groups identified by ASAP (Assemble Species by Automatic Partitioning; *n* = 3 species), SPN (statistical parsimony networks; *n* = 10 species), and PTP (Poisson tree processes; *n* = 10 species). Labels to the right correspond to the ten candidate species groups. The outgroup, represented by *Entosphenus tridentatus*, is not shown.

**Fig 3:**
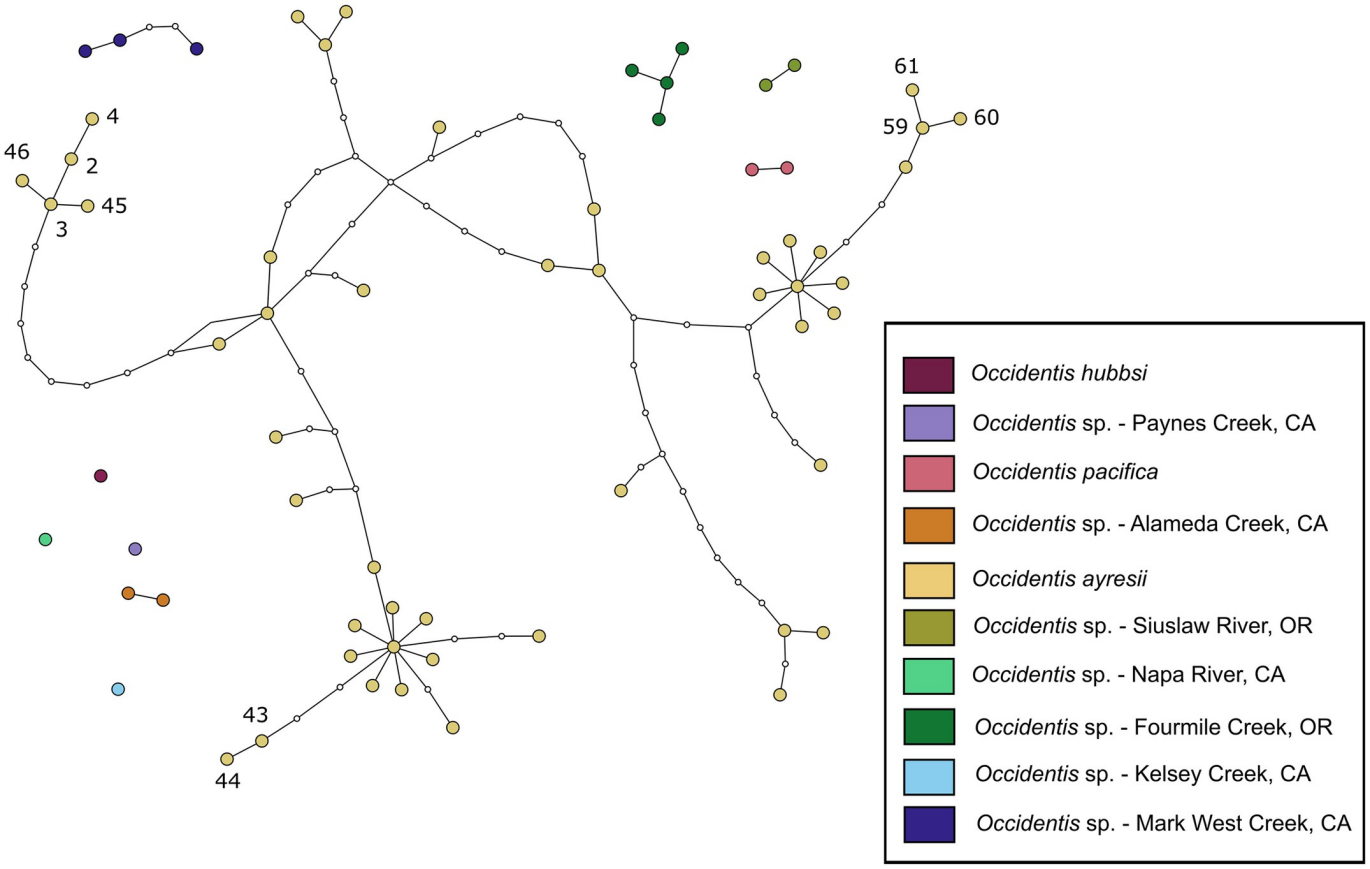
Results of statistical parsimony networks generated from *cyt b* haplotypes of *Occidentis* specimens. Each network displays a candidate species of *Occidentis*; proximity of individual networks does not correspond to relatedness among species. Within a network, colored nodes represent unique haplotypes observed in the current dataset; small white nodes show intermediate haplotypes that were not observed. Segments between nodes represent a single base-pair transition from each connected node. Numbers correspond to haplotypes in S2 Table and highlight lineages of *O*. *ayresii* with high levels of divergence discussed in the main text (Haplotypes 2–4 and 45–46 observed only in subbasins of the Sacramento River, California; haplotypes 43–44 observed only in the Navarro River subbasin, California; and haplotypes 59–61 observed only in the McKenzie River subbasin, Oregon).

Despite differences among methods, results support recognition of 10 candidate species within our dataset. The ASAP analysis supported three candidate species: 1) a lineage from Kelsey Creek (California); 2) a lineage composed of specimens from Alameda Creek, Napa River, and Mark West Creek (California); and 3) a polyphyletic group consisting of all other lineages in the dataset–*O*. *ayresii*, *O*. *hubbsi*, *O*. *pacifica*, and specimens from Paynes Creek (California), Fourmile Creek (Oregon), and Siuslaw River (Oregon) (Fig 2). In contrast, the PTP and SPN analyses delimited ten monophyletic groups of candidate species, including recognized species of *O*. *hubbsi*, *O*. *pacifica*, and *O*. *ayresii*, along with specimens from Kelsey Creek, Paynes Creek, Fourmile Creek, Siuslaw River, Alameda Creek, Napa River, and Mark West Creek (Figs 2 and 3). All ten provisional taxa were reciprocally monophyletic in the maximum-likelihood phylogeny, and six had 93–100% bootstrap support; candidate species from Kelsey Creek, Napa River, Paynes Creek, and *O*. *hubbsi* were represented by a single haplotype and therefore did not receive a bootstrap estimate (Fig 2).

When delimited as ten candidate species, all had minimum interspecific distances ≥ 1.34% (Table 4). Five of six species represented by multiple haplotypes exhibited a maximum intraspecific divergence ≤ 0.49% and a barcode gap. As in Carim et al. [36], *O*. *ayresii* lacked a barcode gap, with the maximum intraspecific divergence (2.43%) exceeding the minimum interspecific divergence with five other species.

**Table 4:** Genetic differences (measured as p-distance and expressed as a percentage) within and among candidate species of *Occidentis*. Values on the diagonal (in bold) are maximum intraspecific differences; values below the diagonal are minimum interspecific differences. Groups with an "--" along the diagonal were represented by a single haplotype, and therefore intraspecific differences could not be estimated.

	Kelsey Creek, CA	*O*. *hubbsi*	Paynes Creek, CA	Fourmile Creek, OR	*O*. *pacifica*	Siuslaw River, OR	Alameda Creek, CA	Napa River, CA	Mark West Creek, CA	*O*. *ayresii*
Kelsey Creek, CA	**--**									
*O*. *hubbsi*	4.62	**--**								
Paynes Creek, CA	5.35	1.34	**--**							
Fourmile Creek, OR	4.99	2.07	2.31	**0.24**						
*O*. *pacifica*	4.99	1.82	2.19	1.95	**0.12**					
Siuslaw River, OR	5.11	2.43	2.68	1.82	1.82	**0.12**				
Alameda Creek, CA	6.08	4.26	4.5	3.89	3.77	4.01	**0.12**			
Napa River, CA	6.81	4.87	5.6	4.99	4.74	5.11	3.04	**--**		
Mark West Creek, CA	6.45	4.50	4.74	3.89	3.89	3.77	1.46	2.80	**0.49**	
*O*. *ayresii*	4.38	1.95	2.19	1.82	1.46	1.70	3.65	4.99	3.77	**2.43**

## Discussion

**Fig 4:**
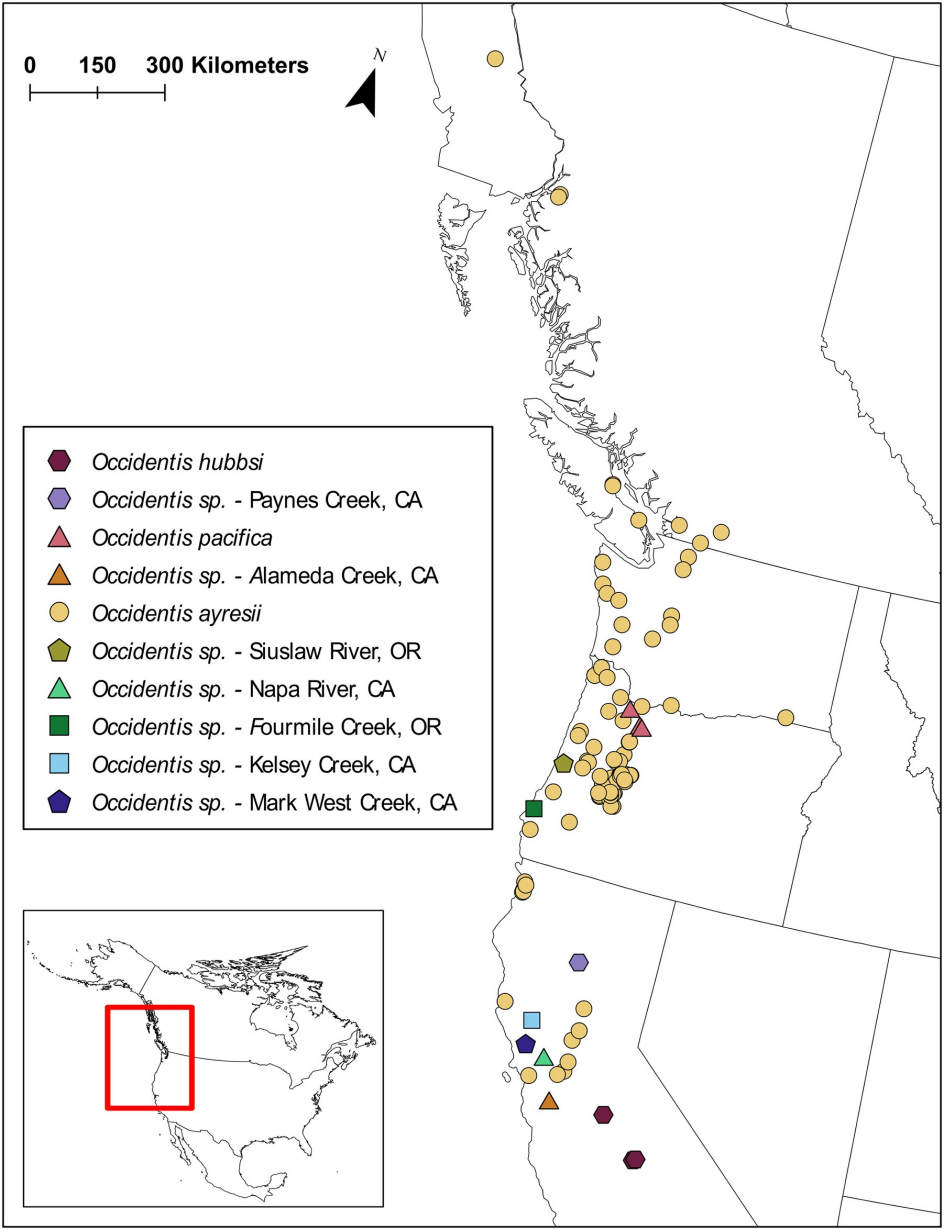
Distribution of candidate species within *Occidentis* identified by species delimitation analysis in this study.

Brook and river lampreys of western North America previously placed in the genus *Lampetra* are genetically distinct from others across the Northern Hemisphere (Fig 1, Tables 2 and 3) and represent an important component of biodiversity that has not been fully appreciated with existing taxonomy [1]. We propose *Occidentis* as a new genus name to classify these brook and river lampreys (*O*. *ayresii* and its junior synonym *O*. *richardsoni*, *O*. *hubbsi*, and *O*. *pacifica*). Note that the taxonomy of brook and river lampreys in the genus *Entosphenus*, including Pit-Klamath brook lamprey *E*. *lethophagus* (Hubbs, 1971) [72], northern California brook lamprey *E*. *folletti* Vladykov & Kott, 1976 [73], and Klamath river lamprey *E*. *similis* Vladykov & Kott, 1979 [74] remains unchanged (see [15]). Although some species of *Occidentis* may show subtle morphological differences from other lampreys, it is geographically widely separated from all members of *Lampetra* and consistently represents a monophyletic group distinct from *Lampetra* at mitochondrial and nuclear genes (see Fig 1; [4, 5, 7–9, 35, 36]), with genetic divergence estimates similar to that observed among other fish genera (Tables 2 and 3; [58]). Similar divergent patterns among genera of Pacific and Atlantic drainages are evident within other migratory fish families—notably the family Salmonidae. For example, trout and salmon of the Pacific drainages were originally classified by Walbaum, 1792 [75] and Richardson, 1836 [63] as *Salmo* Linnaeus, 1758 [18] along with trout and salmon of Atlantic drainages. However, based on thorough review of morphological and ecological similarities (summarized in [76]), all trout and salmon of Pacific drainages were reclassified to *Oncorhynchus* Suckley, 1861 [77, 78], with subsequent support from genetic studies [79, 80].

Similarly, the reclassification of individuals formerly recognized as western North American *Lampetra* to a new genus allows for a more accurate interpretation of evolutionary relationships and better informs efforts to conserve biodiversity of lampreys.

The species delimitation analyses examining diversity within *Occidentis* recovered the three presently recognized species–*O*. *ayresii* (including its junior synonym *O*. *richardsoni*), *O*. *pacifica*, and *O*. *hubbsi*–and six undescribed candidate species from Oregon and California identified by previous studies (Fig 4; [36–38]). The existence of a tenth group as a candidate species remains uncertain. Phylogenetic analysis conducted by Carim et al. [36] and Boguski et al. [38] grouped the specimen from Paynes Creek (California) with *O*. *hubbsi* (although Boguski et al. [38] did not perform a formal species delimitation analysis). In contrast with Carim et al. [36, see also 38], results from both Auringer et al. [37] and this study suggest that *Occidentis* from Paynes Creek may represent a species distinct from *O*. *hubbsi*. The candidate species from Paynes Creek meets the requirement for reciprocal monophyly and was supported as a candidate species by both SPN and PTP analyses in our study. Evidence of a distinct taxon in Paynes Creek is, however, based on a single specimen. *Occidentis hubbsi* is represented by eight individuals from two localities but is also characterized by a single haplotype and exhibits minimal interspecific divergence with the Paynes Creek specimen (1.34%; Fig 4, Table 4). These two lineages were found in separate drainages of the Central Valley in California (*O*. *hubbsi* in the upper San Joaquin River; the Paynes Creek specimen in the upper Sacramento River) and may reflect divergence following colonization of the Central Valley by a shared ancestor. Whether the Paynes Creek specimen represents a distinct taxon or variation within a broader distribution of *O*. *hubbsi* requires further sampling throughout the San Joaquin and Sacramento River drainages followed by phylogenetic analyses.

*Occidentis ayresii* is a broadly distributed species with a high level of intraspecific diversity (Fig 4, Table 4). This species includes some lineages that appear to be endemic to single subbasins and show high levels of isolation by distance, while other haplotypes are broadly distributed across a large geographic range (Figs 2 and 3, S2 Table; see also [36]). For example, haplotypes 59–61 were observed only in the McKenzie River subbasin, a tributary to the Willamette River in Oregon. Lampreys with these haplotypes show <1% divergence from many lineages present in other subbasins of the Willamette River drainage. However, pairwise comparisons of these lamprey to haplotypes 43 and 44 found in the Navarro River (a small river < 50 river km in length draining from the Cascade Mountains off coastal northern California) represent the highest divergence observed among all *O*. *ayresii* in our dataset (2.19–2.43%; Fig 3, S6 Table). Another notable group among the diverging lineages within *O*. *ayresii* are specimens from the Sacramento River drainage (haplotypes 2–4 and 45–46). Auringer et al. [37] regarded this lineage as a candidate species distinct from the rest of *O*. *ayresii*. Indeed, members of this group show the highest level of divergence when compared to the members of the McKenzie River subbasin above (1.95–2.31%; Fig 3, S6 Table), but are as little as 1.09% diverged from other haplotypes in our dataset. Although specimens from these various localities constitute divergent clades within the broader *O*. *ayresii* complex, examining them within the context of specimens from a broad geographic range reveals patterns of relatedness that connect these groups (Fig 3). As a result, the treatment of the Sacramento River drainage lineages as a distinct taxon would result in polyphyly within the *O*. *ayresii* group (Fig 2), violating the phylogenetic species concept. Instead, these broad phylogenetic patterns may be interpreted as lineages undergoing incipient speciation, possibly in response to isolation in glacial refugia during the Pleistocene [81]. Similar patterns occur in other fishes in western North America with similar distributions (e.g., prickly sculpin *Cottus asper* Richardson, 1836; [62, 82]), highlighting the role that geological history has played in shaping the intraspecific diversity of fishes across the region.

Our understanding of inter- and intraspecies diversity in the genus *Occidentis* has rapidly expanded but remains incomplete. Our dataset for species delimitation combined data from several previous studies, using partial sequence data from a single mitochondrial gene. Though single mitochondrial genes are widely used in phylogenetic studies, mitochondrial DNA is inherited maternally and does not recombine; thus, patterns observed at mitochondrial genes may not reflect the complete evolutionary history of a lineage. Future genetic studies investigating diversity in this genus should utilize more widely representative genetic data (i.e. nuclear gene sequences). Despite the broad geographic range of samples in this dataset, there are likely additional unrecognized taxa and lineages, particularly in under-sampled or isolated subbasins and watersheds supporting populations with resident life histories. Further sampling could also determine whether *O*. *pacifica* is endemic to a single river drainage largely lacking natural migration barriers (the Clackamas River subbasin in Oregon) and evaluate the presence of additional cryptic species that may have been overlooked due to similarities with the nonparasitic resident form of *O*. *ayresii* (i.e., western brook lamprey, the former *L*. *richardsoni*; [36]). More information is also needed to determine whether variation observed between groups represents divergent lineages within a species complex (such as observed here in *O*. *ayresii*) or distinct species (e.g., the specimen from Paynes Creek). The number of species formally recognized under *Occidentis* is subject to change with new information. The candidate species identified here are meant to inform—not replace—traditional alpha taxonomy. Spatially comprehensive specimen collection of lampreys, followed by morphological and molecular analyses (using multiple mitochondrial genes and possibly genomic data) will be necessary to validate species diversity in this genus. We strongly encourage biologists from throughout coastal western North America to undertake a systematic, broad-based, and fine-grained assessment of the distribution and phylogenetic complexity of members of *Occidentis*. Such work will enhance our understanding of the evolutionary history and taxonomic diversity of *Occidentis*, which will guide efforts to conserve biodiversity of lampreys.

Management agencies in North America have been developing conservation policies and guidelines for *Occidentis* based on limited available information on species diversity (e.g., [83, 84]). Recognizing intraspecific diversity is imperative for delineating populations that may warrant individualized conservation efforts [1, 85], such as populations exhibiting mixed life histories (e.g., Morrison Creek lamprey in British Columbia, Canada; [86]) or non-migratory populations that have evolved in isolation for many generations (e.g., above geologic barriers). A more complete understanding of intraspecific diversity can promote management actions that foster habitat connectivity (including fish passage), thereby promoting migratory phenotypes and maximizing gene flow among connected populations [84]. Outreach and education will be necessary to clarify that western river lamprey and western brook lamprey are now classified as variants of a single species (*O*. *ayresii*). Similar work will be necessary to clarify that lampreys of western North America formally classified as under *Lampetra* have been reclassified to *Occidentis*.

## Supporting information

S1 Table Diagnostic nucleotides. Cytochrome *b* nucleotide positions diagnostic for the new genus *Occidentis* relative to the genera *Eudontomyzon* (excluding and *E*. *morii*) and *Lampetra* of eastern North America and Eurasia (including *Lethenteron ninae*). Position numbers are those for the GenBank accession Y18683 of *Lampetra fluviatilis*.
(XLSX)

S2 Table Metadata for *Occidentis cyt b* sequences. Metadata for all cyt *b* sequences representing *Occidentis* from western North America analyzed in this study. Under Latitude and Longitude, "NA" indicates that coordinates were not available.
(XLSX)

S3 Table Metadata on additional *COI* and *cyt b* sequences. Information on publicly available sequences used to calculate pairwise genetic distance among genera of Petromyzontidae at the cyt *b* and COI genes. Note that the cyt *b* dataset also included sequences from S2 Table. "Genus group" indicates the genus-level groupings used to generate summary information in Tables 2 and 3. (Citation: Renaud CB, Economidis PS. *Eudontomyzon graecus*, a new nonparasitic lamprey species from Greece (Petromyzontiformes: Petromyzontidae). Zootaxa. 2010;2477: 37–48).
(XLSX)

S4 Table Genus level *cyt b* pairwise p-distance. Pairwise p-distance among n = 536 sequences of Petromyzontidae at 882 bp of the cyt b gene, grouped by genus. Genus groups shown in {}.
(TXT)

S5 Table Genus level *COI* pairwise p-distance. Pairwise p-distance among n = 481 sequences of Petromyzontidae at 585 bp of the COI gene, grouped by genus. Genus groups shown in {}.
(TXT)

S6 Table *Occidentis cyt b* haplotype p-distance. Pairwise genetic distances (p-distances, expressed as percentages) among 66 representative haplotypes of *Occidentis* in this study.
(XLSX)

S1 File Notes on data corrections. (DOCX)
