# Supplementary material for: Species diversity in the new lamprey genus Occidentis, formerly classified as western North American ‘Lampetra’
Source: PLoS One. 2024 Dec 19;19(12):e0313911. doi: 10.1371/journal.pone.0313911 (PMC11658800; doi:10.1371/journal.pone.0313911)
Supplement: S1 File — (DOCX) [file pone.0313911.s007.docx]

**Supplement 1.**

While compiling information for the present study, we identified several errors in Carim et al. (2023). The text erroneously indicates a sample size of n=294 for the *Occidentis cyt b* species delimitation analysis and n=205 additional sequences for species assignment. The actual sample sizes for these analyses were n=275 for species delimitation and n=207 for species assignment as shown in Table S3 of that publication. Figure S1 erroneously omitted a single haplotype of *O. ayresii* from the Long Tom River, OR (GenBank accession ON419582; haplotype 66 in the present study). In Table S3, sample from Fishhawk Creek, OR (GenBank accession GU120787) was mislabeled as sharing a haplotype with a sample from Big River, WA (GenBank accession GU120748); this specimen, in fact, shares a haplotype with another sample from Fishhawk Creek (GenBank accession GU120786). Finally, the candidate species in Fourmile Creek, OR, is located in the Sixes River basin, as indicated in Figure 6 of Carim et al. (2023), not the Klamath River basin as mentioned in the text of that paper. These errors are all minor and did not affect the results or interpretation of this previous study; all have been corrected in the present study.
